# The Roles of E3 Ubiquitin Ligases in Cerebral Ischemia–Reperfusion Injury

**DOI:** 10.3390/ijms26146723

**Published:** 2025-07-13

**Authors:** Man Li, Xiaoxiao Yu, Qiang Liu, Zhi Fang, Haijun Wang

**Affiliations:** 1 Department of Neurology, Union Hospital, Tongji Medical College, Huazhong University of Science and Technology, Wuhan 430022, China; 2 Department of Neurosurgery, Union Hospital, Tongji Medical College, Huazhong University of Science and Technology, Wuhan 430022, China

**Keywords:** E3 ubiquitin ligases, cerebral ischemia–reperfusion injury, mitophagy, inflammation, cell death

## Abstract

The temporary or permanent occlusion of cerebral blood vessels results in ischemic stroke (IS). Ischemia per se causes focal neuronal damage, and the subsequent ischemia–reperfusion injury that occurs after blood flow restoration further compromises brain tissue and cells in the neurovascular unit, significantly contributing to poor patient outcomes and functional impairments. Current research indicates that the ubiquitin–proteasome system (UPS) plays a crucial role in the pathological processes associated with cerebral ischemia–reperfusion injury (CIRI). Notably, E3 ubiquitin (Ub) ligases, which are essential in the UPS, have garnered increasing attention as potential novel therapeutic targets for treating ischemia–reperfusion damage in the brain. This review focuses primarily on the background of E3 Ub ligases and explores their intricate relationships with the pathological processes of CIRI.

## 1. Introduction

As the global population ages, cerebrovascular diseases, particularly strokes, have emerged as the second most prevalent medical condition, posing an ever-increasing threat to humanity [1]. Stroke is classified into ischemic stroke (IS) and hemorrhagic stroke, with IS accounting for as many as 87% of all cases. IS occurs when blood flow to the brain is obstructed due to thrombosis or embolism. Currently, the clinical approach for treating IS focuses on promptly restoring the blood supply to the ischemic area through thrombolytic therapy, which is essential for preserving neuronal vitality and function. However, the reintroduction of blood can lead to an overwhelming production of reactive oxygen species (ROS), which can compromise cellular antioxidant defenses, subsequently triggering inflammatory responses, oxidative stress, mitochondrial dysfunction, and ultimately cell death. This pathological process is known as cerebral ischemia–reperfusion injury (CIRI) [2,3], and the ubiquitin (Ub)–proteasome system (UPS) is involved in this event [4]. Elevated levels of ubiquitin–protein conjugates have been observed in the brain after cerebral ischemia [5] and cerebral-ischemia-induced UPS dysfunction and neuronal injury [6]. However, recent studies have indicated that elevated post-ischemic ubiquitination may result from the suppression of deubiquitinase activity and proteasome inhibition [7,8].

**Figure 1:**
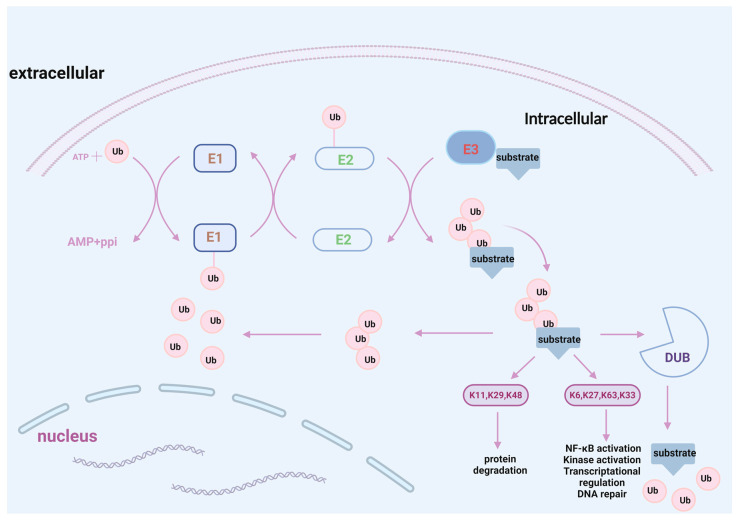
Processes of the UPS.

The post-translational modification (PTM) of proteins is the addition of modifying groups to specific amino acids in a conjugate manner after protein biosynthesis to precisely modulate protein properties and optimize cellular processes [9]. Ub and ubiquitin-like proteins (Ubls) have been identified as the third most common type of PTM after phosphorylation and glycosylation. They maintain cellular protein homeostasis by regulating various biological processes, such as cell cycles, DNA repair, transcriptional regulation, and apoptosis [10,11,12,13]. Among them, Ub, which is composed of 76 amino acids, is a highly conserved protein found in all eukaryotic organisms. In the process of protein ubiquitination, Ub is sequentially added to the protein substrate using Ub-activating (E1), Ub-conjugating (E2), and Ub-ligating enzymes (E3) [14,15]. Subsequently, the ubiquitinated proteins are degraded by the 26S proteasome, while Ub is removed from the substrate by deubiquitinating enzymes (DUBs) and enters the next ubiquitination pathway [16,17]. Ubls are a class of PTM proteins structurally similar to Ub, and their mature form includes the β-grasp domain and a conserved C-terminus with one or two glycine motifs. Similarly to Ub, Ubls are also conjugated to target substrates via the activities of the following three proteins: the E1-activating enzyme, E2 Ubl carrier proteins (Ubcs), and E3 Ubl ligases [18]. The dysregulation of Ubl modifications is associated with various diseases, especially cancer [19]. Ubiquitination occurs through various mechanisms, resulting in different outcomes. For example, monoubiquitination primarily plays a role in nonproteolytic activities (DNA damage response and repair signaling). In contrast, polyubiquitination results in chains with varying biological effects (K6, K11, K27, K29, K33, K48, K63, and M) and is involved in multiple cellular functions [11,20]. In middle cerebral artery occlusion and reperfusion (MCAO/R) mice, the disruption of some polyubiquitin chains attenuates CIRI by suppressing neuroinflammation and protecting neurons [21,22] (Figure 1).

As the critical components of the ubiquitination cascade, E3 ligases strictly control both the efficiency and substrate specificity of the ubiquitination reaction, selecting target proteins by identifying a specific peptide motif termed degrons (short linear motifs) in the substrate [23] and then triggering subsequent downstream signals. Typically, E3 ligases exist in a dormant state to avoid aberrant autoubiquitination or nontarget protein ubiquitination until they are fully activated [24]. Therefore, any alterations in E3 ligase activity can result in changes in Ub-driven pathways, including the proteolytic UPS and protein trafficking and quality control, further affecting fundamental biological processes. Numerous previous studies have shown that E3 ligase dysfunction is associated with the development of many serious diseases, such as cancer, immune disorders, susceptibility to infections, and neurodegenerative diseases [25,26,27,28]. Recent studies suggest that E3 ligases may exert neuroprotective effects during CIRI by modulating the signaling pathways involved in neuronal death, mitophagy, and neuroinflammation [29,30]. Nevertheless, not all E3 ligases show protective effects, as some may exacerbate neural damage by ubiquitinating and degrading protective proteins [31,32], showcasing complicated regulatory mechanisms and diverse biological outcomes.

This review provides insights into the structure, function, and regulation of E3 ligases and uncovers the pathological pathways—such as inflammation, mitophagy, and cell death—in which E3 ligases are involved during the process of CIRI. Exploring the mechanisms of different E3 ligases during CIRI may translate into innovative therapeutic strategies.

## 2. Classification of E3 Ubiquitin Ligases

E3s constitute a vast family of enzymes involved in the ubiquitination process, regulating the activity of various proteins. Currently, more than 600 distinct E3s have been identified. According to their E2 binding domain structure and ubiquitin transfer mechanism, the superfamily can be broadly categorized into the following three classical families: the RING (Really Interesting New Gene) finger family, the HECT (Homologous to the E6AP-Carboxy Terminus) family, and the RBR (RING Between RING) family [12,25].

### 2.1. RING Finger Family E3 Ligases

The RING finger E3 ligases constitute the largest E3 family, and their activity depends on their RING finger domain or U-box catalytic domain. The canonical RING finger is a cysteine-rich domain bearing two zinc ions, and it can directly mediate the transfer of ubiquitin from bound E2 (E2-Ub) to the target substrate [33,34]. The RING E3 ligases can be classified into five distinct subfamilies, all of which share a similar N-terminal RING domain with various unique domains [33]. Furthermore, RING E3 ligases can be divided into monomeric, homodimeric/heterodimeric, and multisubunit forms. Cullin-RING E3 ligases (CRLs), which constitute the largest subgroup of RING E3 ligases, account for 20% of all cellular ubiquitination [35]. In general, CRLs consist of the following four components: cullin scaffold proteins, E2 binding RING-box proteins (Rbx1 and Rbox2), adaptor proteins, and substrate recognition proteins [36]. Differences in cullin types (Cul1, Cul2, Cul3, Cul4A, Cul4B, Cul5, Cul7, and Cul9) form the basis for different groups of the CRL subfamily [37]. Meanwhile, the SCF (SKP1-Cullin-F-box) E3 ligases constitute the largest CRL family, with SKP1 as the adaptor and F-box proteins as the substrate recognition unit [38]. The human genome contains 69 F-box proteins, which are crucial regulators of diverse cell functions and can be classified into the following three subfamilies according to their substrate-recruiting domains: F-box with the WD-40 domain (FBXW), F-box with the leucine-rich repeat (FBXL), and F-box with other domains (FBXO) [39,40].

The cell cycle is a tightly orchestrated cellular process and is primarily driven by the sequential activation of cyclin-dependent kinases (CDKs). Cyclin partners and CDK inhibitors (CKIs), which are tightly controlled by the UPS, can modulate the activity of CDKs [41]. In eukaryotic cells, anaphase-promoting complex/cyclosome (APC/C) and SCF E3 ligases are mainly responsible for the ubiquitination and proteasomal degradation of these CDK regulators [42]. APC/C, a multi-subunit cullin-RING E3 ubiquitin ligase, functions in the mitotic phase and G1 phase, regulating cell cycle progression through the M phase and entry into the S phase [43]. The function of the SCF E3 ligase complex in the cell cycle is more complex; thus, it is not elaborated in detail here [44]. Overall, cell cycle progression is tightly regulated, whereas during cerebral ischemia, the dysfunction of the ubiquitination modification will cause uncontrolled cell cycle progression and may promote neuronal and glial cell death.

It is worth emphasizing that cullin proteins are the best-recognized substrates for protein neddylation. Neddylation, as a reversible post-translational modification [45], adds a Ubl, NEDD8 (neuronal precursor cell-expressed developmentally downregulated protein 8), to targeted substrate proteins via a three-step enzymatic cascade catalyzed by NEDD8-activating enzyme E1 (NAE), NEDD8-conjugating enzyme E2s (UBC12/UBE2M or UBE2F), and substrate-specific NEDD8-E3 ligases [46]. Cullin neddylation can result in the activation of CRLs, which then ubiquitinate cellular proteins to degrade via the UPS [47,48]. Currently, accumulating evidence indicates that targeting neddylation is a promising therapeutic strategy because many key proteins in neddylation can be activated in diseases such as cancer, fibrotic diseases, cardiovascular diseases, etc. Interestingly, in transient focal ischemia mice, studies have found that neddylation is upregulated in the brain and active in intravascular and intraparenchymal neutrophils. ML4924, a neddylation inhibitor, can reduce both neutrophil extravasation and BBB breakdown through the attenuation of NEDD8 conjugation to cullin-1, ultimately reducing BBB permeability after cerebral ischemia and exhibiting neural protective effects [49]. Additionally, CRLs can regulate autophagy by targeting ATGs and upstream regulators such as MTORC1 [50], and they involve important inflammation-associated pathways such as NF-κB, JAK/STAT, and TGF-β [38].

Recently, many studies have demonstrated that the RING finger E3 ligase family participates in the process of CIRI. For example, FBXO3, the substrate recognition subunit of SKP1-cullin 1-F-box protein (SCF) E3 ligase complexes, is significantly elevated in the peri-infarcted brain tissue of SD rats subjected to MCAO/R and specifically expressed in neurons, which can ubiquitinate and degrade HIPK2 (a convincing anti-inflammatory cytokine) to accelerate neuroinflammation and aggravate CIRI [31]. In contrast, RNF8 holds potential neuroprotective properties against ischemic stroke through the HDAC2/Reelin/GSK3β axis [30]. Other RING E3 ligases involve different mechanisms of CIRI; thus, they are described below.

#### 2.1.1. TRIM E3 Ligases

The tripartite motif (TRIM) family is one of the largest classes of putative single-protein RING-finger E3 ligases and includes an N-terminal RING domain, one or two B-box motifs, an alpha-helical coiled-coil domain, and a highly variable carboxy-terminal domain [51]. The RING domain constitutes the catalytic center and is involved in ubiquitylation pathways such as the recruitment of Ub-conjugating enzymes, while the B-box motifs contain finger-like protrusions that participate in the recognition of target proteins. TRIM proteins often form large protein complexes by self-associating through their coiled-coil regions, eventually settling in the cytoplasm or nucleus. They can be classified into 11 unique subgroups on the basis of their highly variable carboxy-terminal domain [52], and these variable domains constitute the functional units that mediate target recognition and specificity.

TRIMs may act as receptors or scaffold proteins that direct substrates for autophagy-related degradation [52]. Indeed, TRIM proteins might also play a “nonproteolytic” role in ubiquitination. Current studies indicate that TRIM proteins represent a novel class of small Ub-like modifier (SUMO) E3 ligases. SUMO is a type of post-translational modification that is similar to the ubiquitination process, which usually changes the properties of target proteins, such as their stability, activity, or cellular localization [53]. Meanwhile, the TRIM family is involved in various cellular processes, such as apoptosis, viral response, cell proliferation, cellular cycles, etc. TRIM proteins can act on crucial factors, such as the P53 or JAK/STAT signaling pathways, for optimal cell cycle progression or cell proliferation [54]. However, these dysfunctions cause cell cycle arrest and are involved in human diseases.

During the pathological process of CIRI, different inflammatory pathways are activated to release large amounts of proinflammatory mediators, such as IL-1β, IL-6, and TNF-α, exacerbating damage to neighboring neurons and resulting in the delayed deterioration of ischemic tissue. The TRIM protein family appears to be involved in this signaling pathway. Suppressing TRIM62 or TRIM8 could inhibit neuronal apoptosis and neuroinflammation through mitigating the NLRP3/NF-κB signaling pathway during CIRI [55,56]. Recently, the TRIM family has become a trending research topic, and the inhibition of the TRIM family may be a new strategy for the treatment of CIRI.

#### 2.1.2. U-Box E3 Ligases

U-box-type E3 ligases are a subgroup of RING-type E3 ligases that can act as monomers or homodimers. The C-terminus of U-box-type E3 ligases contains a conserved U-box domain consisting of approximately 70 amino acids. This domain interacts with Ub-conjugating E2 enzymes to directly transfer Ub molecules to target proteins [20]. Among various U-box-type E3 ligases, such as well-known U-box-type E3 ligases and co-chaperones, CHIP is responsible for ubiquitination and can modulate multiple cellular signaling pathways linked to the pathophysiology of CIRI [57]. Additionally, UFD-2 is an important U-box E3 ligase. With the assistance of partner proteins, UFD-2 can facilitate the polyubiquitination of unfolded myosin, playing a crucial role in maintaining protein homeostasis in muscle cells [58].

### 2.2. HECT Family E3 Ligases

The human proteome contains 28 distinct HECT-type E3 ligases. Each of these ligases features a C-terminal HECT domain composed of N and C lobes, which weigh approximately 40 kDa. HECT-type E3s promote ubiquitination in a two-step process: the N lobe specifically recognizes substrates and interacts with E2 enzymes, after which Ub is transferred from E2 enzymes to an active cysteine site on the C lobe to label target proteins for ubiquitination [59,60]. HECT-type E3 enzymes are further categorized into three families, the Nedd4 family, the HERC family, and other HECT-type ligases, based on the domain architectures of their N-termini. Among them, NEDD4, a founding member of the NEDD4 family, was originally thought to be a developmental regulatory gene in the CNS and regulates dendritogenesis and neuronal polarity [61,62]. The HERC subfamily only has six members, is mainly located in the cytoplasm, and regulates MAPK signaling pathways to contribute to cancer and neurodevelopmental diseases [63]. Other HECT-type ligases, such as the Ub ligase E6-associated protein (E6AP), are expressed in neurons and glial cells, and their abnormal activity may also contribute to the development of various brain diseases, such as PD, Huntington’s disease, and Alzheimer’s disease [64]. HECT family E3 ligases help to protect neurons and glial cells from oxidative stress in CIRI.

### 2.3. RBR Family E3 Ligases

The RBR ligases constitute the smallest E3 family, which only has 14 members, and some well-known E3 ligases, such as Parkin, HOIP, HHARI, RNF144, and TRIAD1, are also included. The RBR ligases are composed of the following three components: RING1 with an E2 binding domain, RING2 with a catalytic cysteine residue, and an In-Between Ring (IBR) domain. Similar to the HECT-type E3s, RBR ligases promote the transfer of ubiquitin from an E2 to the second RING domain of RBR E3s—creating a thioester intermediate—before facilitating transfers from E3s to the substrate [65]. Traditional RBRs exist in the form of N-RING1-IBR-RING2-C, but they can contain additional domains that confer a characteristic auto-inhibitory action to the RBR family [24]. For example, Parkin, an RBR-type tumor suppressor linked to neurodegenerative disorders and the innate immune response, remains in an autoinhibitory state via the following three outside domains: the Ub-like domain (Ubl), the Ring domain, and an REP domain. The phosphorylation of the Ubl domain by PTEN-induced kinase (PINK1) and the binding of phosphorylated Ub to Parkin disrupt the autoinhibitory state, releasing Parkin for activity [66,67].

Several RBR proteins can influence the expression of key factors in related signaling pathways by modulating the stability and activity of proteins associated with cell growth and signaling [68]. Additionally, dysfunctions in RBR proteins have been linked to the development of CIRI. A prime example is Parkin, which interacts with PINK1 to direct damaged mitochondria to degradation during mitophagy. This process is important for mitigating cerebral ischemic damage [29].

## 3. Roles of E3 Ligases in CIRI

The UPS is essential for neuron development through embryogenesis and proteostasis maintenance during adulthood. Neurons undergo vast proteome turnover during differentiation, moderated in part through ubiquitin-mediated proteasomal degradation. In the terminally differentiated neurons of neurodegenerative disease, misfolded proteins and protein aggregates are partially cleared out of the cell due to ubiquitin signaling. Ubiquitination is a dynamic and highly reversible PTM conferred by E3 ubiquitin ligases, and the normal expression and activation of E3s are particularly important. Previous studies regarding E3 ligases have focused on cancer, neurodegenerative diseases, and neurodevelopmental disorders [25]. For example, the impairment of Parkin’s E3 ligase activity is believed to play a pathogenic role in both familial and sporadic forms of PD [69]. The HECT E3 ligase Nedd4 subfamily is directly or indirectly involved in neurodevelopmental diseases such as Alzheimer’s disease and Amyotrophic Lateral Sclerosis [70]. Abnormal HECT E3 ligase E6AP activity contributes to schizophrenia, Huntington’s disease, Alzheimer’s disease [71], etc. However, it has been found that some E3 ligases are involved in the ischemia–reperfusion process. For example, FBXO3 can drive neuroinflammation to aggravate CIRI [31]. The E3 ligase MARCH1 reduces inflammation and pyroptosis in CIRI via PCSK9 downregulation [72]. PA2G4/EBP1 ubiquitination via PRKN/PARKIN promotes mitophagy, protecting neurons from death in cerebral ischemia [29], etc. Based on the above research background, we screened the key members of the RING, HECT, and RBR E3 ligase families and found that these protein molecules promote or inhibit ischemic stroke injury (Table 1) by regulating the signaling pathways of neuroinflammation, mitophagy, and cell death [1,2,3].

### 3.1. Neuroinflammation

Inflammation is a significant contributor to CIRI. IS activates a variety of cells, such as microglia and astrocytes [99]. These activated cells subsequently release large amounts of inflammatory mediators, such as TNF-α, interleukin-1β (IL-1β), and interleukin-6 (IL-6), aggravating brain injury [100,101]. Furthermore, impaired brain cells can release various cellular components, such as damage-associated molecular patterns (DAMPs), subsequently activating immune cells and eliciting inflammation [102].

In the classical inflammatory pathways activated by CIRI, TLRs are activated by DAMPs, and then adaptor molecule myeloid differentiation factor 88 (MyD88) is recruited, which subsequently engages the E3 ligase TNF receptor-associated factor 6 (TRAF6). The activated TRAF6 interacts with IKKα/β/γ, TAK1, and the RIPI complex through autoubiquitination, ultimately resulting in the activation of NF-κB and the release of proinflammatory cytokines [103]. The nonclassical pathway involves signaling molecules such as BAFF, CD40L, RANKL, and LTβ, which activate their receptors and subsequently interact with NIK to phosphorylate IKKα and downstream RelB, ultimately activating NF-κB. Additionally, various other complex signal transduction pathways, including the PI3K/AKT, MAPK, JAK-STAT, and WNT pathways, can participate in the NF-κB pathway and induce neuroinflammation [103].

The RING finger (RNF) family, characterized by the N-terminal RING structural domain, is involved in many biological processes, contributing to several diseases, such as cancer, immunological diseases, and neurological disorders [104]. Research has indicated that RNF proteins play a vital role in regulating inflammation signaling pathways. The RNF41 protein, also known as Nrdp1, directly interacts with MyD88, promoting its degradation via K48-linked polyubiquitination, negatively regulating the MyD88-mediated activation of NF-κB and AP1, and suppressing the production of proinflammatory cytokines [74]. RNF56 (Cbl-b) directly interacts with MyD88, triggering its degradation via polyubiquitination, which effectively inhibits MyD88-mediated inflammatory responses [75]. Conversely, RNF152 associates with the adaptor protein MyD88, enhancing the oligomerization of MyD88 and positively regulating the TLR/IL-1R signaling pathway to promote inflammation [77]. The E3 ligase TRAF6 ubiquitinates and activates RAC1 to promote neuroinflammatory and neuro-oxidative signaling, exacerbating neuronal death. In mouse experiments, TRAF6 knockdown attenuated CIRI, whereas TRAF6 overexpression aggravated injuries [96].

RNF proteins also orchestrate the activation and stability of NF-κB. The proinflammatory cytokines TNFα and IL-1β initiate the phosphorylation and proteasomal degradation of IkB, which activates the release of NF-κB from IkB. NF-κB subsequently migrates into the nucleus, where it releases proinflammatory cytokines such as NOS, TNF-α, and IL-1, resulting in neuronal damage. RNF114 negatively regulates NF-κB signaling by promoting the ubiquitination and stabilization of IκBα, which inhibits NF-κB-dependent transcription [73]. In contrast, RNF121, an E3 ligase tethered to the Golgi apparatus, facilitates the proteasomal breakdown of IκBα to positively regulate the activation of the NF-κB signaling pathway [76]. In addition to the RNF family, the TRIM family also participates in the NF-κB signaling pathway. In the cytoplasm, TRIM8 activates the NF-κB signaling pathway via TNFα and IL-1β. In the nucleus, TRIM8 facilitates the translocation and degradation of the protein inhibitor of the activated STAT3 (PIAS3), preventing the activation of NF-κB. Furthermore, TRIM8 promotes the Lys63-linked polyubiquitination of TAK1, leading to IKK kinase activation and resulting in the phosphorylation of IκBα and the subsequent translocation of NF-κB [60,84]. During CIRI, the overexpression of TRIM8 in microglia triggers the release of proinflammatory cytokines, provoking a substantial neuroinflammatory response and resulting in neuronal injury and brain damage [60]. TRIM45 directly interacts with TAB2 by promoting the K63-linked polyubiquitination of TAB2, subsequently recruiting TAB1/TAK1, inducing the formation of the TAB1–TAK1–TAB2 complex and facilitating the autophosphorylation of TAK1. The activated TAK1 subsequently triggers NF-κB-mediated inflammation and neuronal cell death during CIRI [33]. Recent studies have indicated that TRIM47 knockdown can hinder the NF-κB signaling pathway in brain samples from middle cerebral artery occlusion (MCAO) rats, apparently decreasing the release of proinflammatory factors such as IL-6 and TNF-α [87]. Interestingly, TRIM9 appears to be unique and can sequester the β-transducin repeat-containing protein (β-TrCP) from the Skp-Cullin-F-box ligase complex to hamper the degradation of IκBα, thereby dampening NF-κB-dependent proinflammatory mediator production and immune cell infiltration [85].

Inflammasomes are multiprotein complexes formed upon cellular infection or stress, regulating the maturation of proinflammatory cytokines, such as IL-1β and IL-18, and subsequently triggering inflammation and immune defense [105]. Most inflammasome receptors are nucleotide-binding leucine-rich repeat receptor (NLR) sensors, including NLRP1, NLRP3, and NLRC4, with NLRP3 being the most characterized among them. Canonical NF-κB activates the expression of NLRP3 and pro-IL-1β in response to stimulation by microbial components or cytokines, serving as an NLRP3 priming signal [106]. TRIM62 knockout reportedly represses the NLRP3 inflammasome, subsequently restraining neuroinflammation during CIRI [55]. Silencing TRIM22 can relieve oxygen–glucose deprivation/reoxygenation (OGD/R)-induced apoptosis and inflammation by inhibiting the NF-κB/NLRP3 axis [87]. Moreover, TRIM29 can directly interact with NLRC4 to promote the K48-linked polyubiquitination of NLRC4. This process results in the proteasomal degradation of NLRC4, effectively suppressing NLRC4 inflammasome activation and alleviating neuroinflammation caused by CIRI [95].

**Figure 2:**
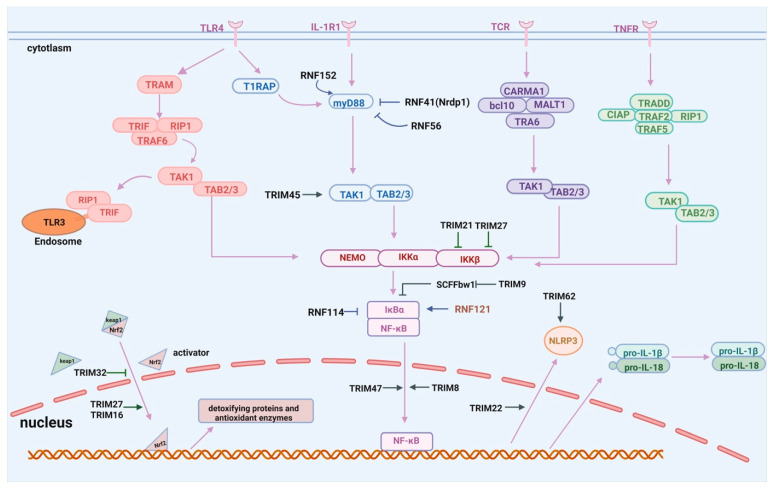
Classic members of the E3 ligase family participate in modulating the neuroinflammatory pathways triggered during the process of CIRI.

Under normal conditions, the transcription factor nuclear factor erythroid 2-related factor 2 (NRF2) is retained in the cytoplasm via Kelch-like ECH-associated protein 1 (KEAP1) [107]. However, oxidative stress during I/R injury disrupts this complex and activates NRF2. Once activated, NRF2 translocates into the nucleus, where it binds to the antioxidant response element (ARE) in the upstream promoter regions of many antioxidative genes, regulating the expression of multiple antioxidant enzymes, including heme oxygenase-1 (HO-1), SOD, catalase (CAT), and NAD(P)H quinone oxidoreductase-1 (NQO-1) [108]. The knockdown of TRIM32 has a protective effect on oxidative damage in hippocampal neurons induced by OGD/R through the activation of the NRF2 signaling pathway [86]. TRIM27 overexpression activates the Akt/NRF2/HO-1 pathway in MCAO/reperfusion (MCAO/R) mice, inhibiting inflammation [88]. Furthermore, TRIM16 overexpression provides a cytoprotective effect against OGD/R-exposed neurons by enhancing NRF2/ARE antioxidant signaling via the downregulation of Keap1 [89] (Figure 2).

### 3.2. Mitophagy

Cerebral ischemia is a leading cause of mortality and disability. Mitochondria are crucial organelles that are primarily responsible for energy production. However, in the first hours following ischemia, the functional integrity of the mitochondrial membrane is compromised; damaged mitochondria release harmful ROS and other oxidants, such as H2O2 and peroxynitrite, into the cytoplasm, inflicting damage to proteins, nucleic acids, and cell membranes [109]. Simultaneously, damaged mitochondria also cause the release of CYCS (cytochrome c), the activation of caspases, and apoptosis [110]. Mitophagy is an important mechanism that eliminates damaged mitochondria and thereby protects neurons against ischemic injury [111]. Thereby, the stimulation of mitophagy may hold promise as a potential therapeutic strategy against CIRI.

There are three main mitophagy pathways in mammalian cells. These include the PINK1 (PTEN-induced putative kinase 1)-PRKN/PARKIN (a RING/HEC1 type E3 ligase)-mediated pathway and two PRKN-independent pathways, including the BNIP3L/NIX (BCL2 interacting protein 3-like)-BCL2 (BCL2 apoptosis regulator)-BNIP3 (BCL2 interacting protein 3) pathway and FUNDC1 (FUN14 domain containing 1) pathway [112]. The dysfunction of PINK1-PRKN-dependent mitophagy is associated with CIRI.

The PINK1-PRKN/PARKIN pathway includes mitochondrial, depolarized, or accumulating misfolded membrane proteins. When PINK1 phosphorylates itself and is located on the outer mitochondrial membrane, phosphorylated Ub can directly bind to Parkin and is recruited to the surface of mitochondria and phosphorylated via PINK1. Subsequently, mitochondria labeled with Ub attract mitophagy receptors such as p62, NDP52, NBR1, and OPTN. These receptors interact with the autophagy protein LC3 on the outer membrane through their LC3-interacting region (LIR) motifs and bind to polyubiquitin chains via their Ub-binding domain, ultimately initiating mitophagy [111,113].

The PINK1-PRKN-dependent mitophagy pathway has become a trending topic of research in recent years. PA2G4/EBP1 serves as a crucial regulator of neuronal survival, differentiation, and axon regeneration after injury, contributing to multiple types of cellular signaling [114,115]. Moreover, PA2G4/EBP1 has been identified as a neuronal substrate of the PRKN E3 ligase. K63-linked ubiquitination mediated by PRKN on PA2G4/EBP1 can recruit the adaptor protein SQSTM1, thereby inducing mitophagy to provide neuroprotective effects. Additionally, research has indicated that PA2G4/EBP1 expression increases within 24 h in the hippocampus after ischemic damage, preventing neuronal death, decreasing brain infarct volume, and alleviating motor and cognitive impairments [29]. Adenylate kinase 4 (AK4) is an adenylate kinase isoenzyme that is a family of ubiquitous enzymes involved in high-energy phosphorylated transfer in living cells, and it is involved in cytoplasmic, mitochondrial, and nucleotide energy metabolism in the mitochondrial matrix [95]. Research has demonstrated that AK4 increases the interaction between pyruvate kinase M2 (PKM2) and its E3 ligase Parkin to increase PKM2 ubiquitination, indirectly decreasing PKM2 expression [116]. PKM2 serves as a crucial mediator of cellular energetics and may aggravate neuroinflammation after CIRI. These data suggest that the AK4/Parkin/PKM axis prevents cerebral ischemia damage by regulating neuronal energy metabolism and mitophagy.

The BNIP3L/NIX-BCL2-BNIP3 pathway involves the outer mitochondrial membrane (OMM) protein BNIP3L and its analog NIX, which are considered apoptotic proteins. LIR motifs can directly facilitate interactions between mitochondria and LC3, resulting in their binding to the polyubiquitin chain through the Ub-binding domain, which induces mitophagy. Furthermore, NIX/BNIP3 can induce mitophagy by interacting with the homolog of autophagy-related gene protein 8 (Atg8) through its LIR motif. NIX/BNIP3 can also mediate mitophagy under hypoxia by stabilizing HIF1α [117]. Currently, NIX/BNIP3-mediated mitophagy has been confirmed to alleviate CIRI. Specifically, cerebral ischemia can lead to extensive mitochondrial damage. However, the overexpression of BNIP3L within the BNIP3L/NIX pathway can trigger an increase in mitochondrial autophagy, thereby eliminating damaged mitochondria and contributing to neuroprotection [118].

Under hypoxic conditions, FUN14 domain-containing 1 (FUNDC1)—another OMM protein that contains a transmembrane LIR motif—can be activated. Dephosphorylated FUNDC1 induces mitophagy by interacting with LC3. These data suggest that FUNDC1 plays a pivotal role in ischemic injury. Tissue plasminogen activator (tPA) reportedly responds to oxidative stress during IR by increasing the phosphorylation of AMPK, increasing glucose uptake in neurons, and promoting mitochondrial ATP production [119]. tPA can also improve mitochondrial function and reduce neuronal apoptosis by increasing the expression of FUNDC1 [120]. Additionally, pleckstrin homology-like domain family A member 1 (PHLDA1) can regulate oxidative stress, immune responses, and apoptosis. In OGD/R-treated primary hippocampal neurons, silencing PHLDA1 was found to increase mitophagy by activating FUNDC1 to mitigate neuronal damage [121].

Currently, several mitophagy-related drugs are attracting attention. Ligustilide (LIG) reportedly enhances mitophagy via the PINK1/Parkin pathway and ameliorates neuronal injury during IS [122]. The histone deacetylase sirtuin 1 (SIRT1), a crucial regulator of mitochondrial autophagy, activates the PINK1/Parkin signaling pathway. P-hydroxybenzyl alcohol (pHBA) has been reported to mediate the SIRT1 pathway to promote mitophagy and, thus, exert neuroprotective effects [123]. Additionally, active components of Polyrhachis vicina (Roger) alleviate CIRI by activating the PINK1/Parkin signaling pathway [124]. The degradation of the mitophagy receptor BNIP3L/NIX by proteasomes results in mitophagy deficiency [125].

### 3.3. Cell Death

**Figure 3:**
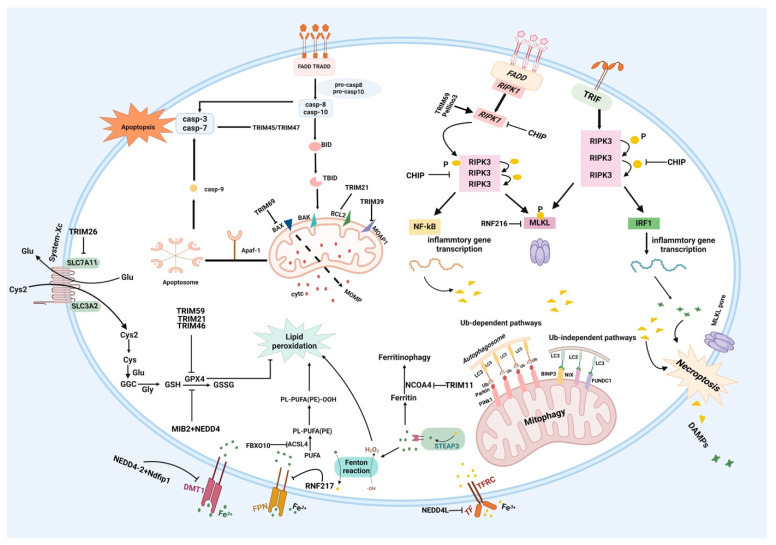
Classic members of the E3 ligase family participate in regulating neuronal death, including mitophagy/apoptosis and necroptosis/ferroptosis, during the process of CIRI.

Under CIRI, when blood supply is restored after the interruption of cerebral blood flow, local neuroinflammation and excessive reactive oxygen species (ROS) cause secondary brain damage. Multiple cell death mechanisms, including apoptosis, necrosis, necroptosis, autophagy, pyroptosis, and ferroptosis, are implicated in this process. E3 ligases participate in the cell death mechanisms associated with the pathological process of CIRI [126,127] (Figure 3).

#### 3.3.1. Apoptosis

Apoptosis is a type of caspase-dependent programmed cell death. Apoptotic signal transduction involves the following two main pathways: the intrinsic pathway (mitochondria-mediated apoptosis) and the extrinsic pathway (death-receptor-mediated apoptosis) [128].

Mitochondria primarily mediate the intrinsic pathway. Intrinsic stimuli arising from cellular stress and DNA damage induce the expression or activation of BH3-only proteins, activating Bax or other proapoptotic proteins to form pores in the outer mitochondrial membrane and inhibiting complex II and ROS production. Cytochrome c is subsequently released, activating the OMA-1 protease and restructuring the inner mitochondrial membrane. The released cytochrome c binds to APAF-1; activates caspase-9; leads to the cleavage of caspase-9 and the activation of downstream caspases; and induces mitochondrial outer membrane permeabilization (MOMP) [129]. Antiapoptotic proteins, such as Bcl-2 and Bcl-xL, antagonize the formation of MOMP. The transcription factor p53, a proapoptotic regulator, targets the promoter regions that control the expression of several proapoptotic BCL-2 proteins. Proapoptotic proteins, including Bax and Bad, prompt MOMP to release downstream-signaling caspase-activating proteins, cytochrome c, and second mitochondria-derived activator of caspases (SMACs), ultimately inducing apoptosis. This intricate regulatory system ensures precise control of the apoptosis process [129]. With respect to the extrinsic apoptosis pathway, the TNFR family of death receptors (DRs) recruits death domains such as TRADD and FADD to facilitate intracellular signaling and activate caspase-8. Once caspase-8 is activated, it either cleaves downstream caspases or activates the BH3-only protein Bid, inducing MOMP [130].

Apoptosis plays a crucial role in the progression of cerebral ischemia. Several TRIM proteins may be involved in the signaling pathways associated with apoptosis [56]. TRIM21, a universal proapoptotic protein, induces apoptosis by targeting the antiapoptotic protein BCL2 through p53 [91]. An increased expression of TRIM69 can induce the expression of apoptosis regulators such as Bax to promote apoptosis [56]. TRIM16 induces apoptosis by directly binding to caspase-2 and modulating its activity [92]. TRIM39 can inhibit the polyubiquitination and degradation of the modulator of apoptosis 1 (MOAP1) to increase its levels in mitochondria, thus regulating apoptosis [131]. TRIM27 plays a role in the apoptotic signaling pathways of MAPKs and caspases and also positively regulates TNF-induced apoptosis [93]. TRIM32 initiates TNF-α-induced apoptosis. The knockdown of TRIM45 in microglia reverses the overexpression of cleaved caspase-3, caspase-9, and PARP in neurons exposed to OGD/R, exerting an antiapoptotic effect on neurons [33]. TRIM47 acts as a proapoptotic regulator in the MCAO model. The knockdown of Trim47 alleviates cerebral ischemic injury by reversing the increase in cleaved caspase-3 and the reduction in the expression of Bcl-2 [87]. In addition to the already discussed TRIM proteins, several other TRIM proteins that are localized in the nucleus, such as TRIM19, TRIM17, and TRIM37, participate in apoptosis signaling pathways via different mechanisms, but their specific functions in the process of CIRI remain unclear [81].

#### 3.3.2. Necroptosis

Necroptosis—known as programmed necrosis—is recognized as one of the various forms of cell death following CIRI. Necroptosis following CIRI is induced by energy depletion, oxidative stress, and ROS accumulation. Cellular organelle swelling and cell membrane rupture are the morphological features of necroptosis [127].

During the process of necroptosis, activated death domain receptors (e.g., TNFR and Fas) and Toll-like receptors recruit the adapter proteins FADD, TRADD, and TRIF, which subsequently interact with RIPK1. Usually, RIPK1 remains nonfunctional because it is ubiquitylated by the inhibitors of apoptosis (IAPs). When a “death signal” is detected, the Ub carboxyl-terminal hydrolase (CYLD) deubiquitylates RIPK1 to recruit RIPK3. The RIPK1/RIPK3 complex then recruits and phosphorylates mixed-lineage kinase domain-like pseudokinase (MLKL). Phosphorylated MLKL oligomerizes in the presence of highly phosphorylated inositol phosphate (IP6), forming the necrosome. MLKL oligomers translocate to the plasma membrane and form large pores, which result in necroptotic cell death by allowing ion influx, cell swelling, and membrane lysis [128,130].

Several studies have shown that inhibiting necrotic apoptosis can reduce cerebral infarct volume and improve motor and cognitive function in MCAO/R mice. In recent years, the function of E3 ligases in necroptosis has attracted increasing attention. The carboxyl terminus of Hsp70-interacting protein (CHIP) is an E3 ligase with a molecular chaperone that participates in cellular protein quality control by modulating the degradation of chaperone-bound proteins [57]. The formation of phosphorylated RIPK3 (p-RIPK3) and phosphorylated MLKL (p-MLKL) is a crucial stage in necroptosis progression, while CHIP can negatively regulate necroptosis by increasing the degradation of RIPK1 and RIPK3 to alleviate CIRI [57]. RNF216, also known as Triad3A, is an E3 ligase containing a RING finger that can regulate the degradation of RIPK3-interacting proteins, thereby controlling necroptosis and the expression of inflammatory cytokines [78]. RNF216 has been reported to suppress necroptosis following IS through interactions with MLKL [83]. Pellino3, an E3 ligase, can prevent the formation of death-induced signaling complexes by targeting RIPK1 [105].

#### 3.3.3. Ferroptosis

Intracellular iron overload and the overaccumulation of reactive oxygen species (ROS)—caused by iron metabolism disorders—are hallmarks of ferroptosis, which plays an essential role during CIRI [132]. Mechanistically, Fe^3+^ is released from transferrin in the acidic environment of endocytosed vesicles and is reduced to Fe^2+^. Fe^2+^ then enters the labile iron pool in the cytoplasm through divalent metal transporter 1 (DMT1) and can be reoxidized to Fe^3+^ and exported to the extracellular space by membrane iron transporter 1. Excess iron can be stored in ferritin. This intricate process maintains iron metabolism and achieves intracellular iron homeostasis. However, disrupted iron metabolism results in the production of toxic hydroxyl radicals, generating highly reactive ROS via the Fenton reaction. ROS can interact with the polyunsaturated fatty acids (PUFAs) found in cell membrane lipids, initiating lipid peroxidation. Furthermore, acetyl-CoA synthetase long-chain family member 4 (ACSL4) and lysophosphatidylcholine acyltransferase 3 (LPCAT3) can activate PUFAs and increase their association with membrane lipids, subsequently increasing cellular peroxidative damage and contributing to ferroptosis. Lipoxygenase (LOX) also mediates ferroptosis by facilitating lipid peroxidation [133,134,135,136,137,138,139]. Ferritinophagy involves the formation of autophagic vesicles promoted by NCOA4, and autophagic vesicles transport ferritin to the lysosome for degradation, which releases large amounts of free iron and causes disturbed iron metabolism and ferroptosis [132,133].

The modulation of E3-associated signaling pathways attenuates ferritinophagy and ferroptosis after cerebral ischemia/reperfusion, thus exerting protective effects on neurons.

NEDD4L is a constituent element of the HECT E3 ligase family. m6A methyltransferase (METTL3) overexpression in mouse stroke models increases NEDD4L levels, and NEDD4L reduces intracellular iron accumulation through the ubiquitination and degradation of transferrin receptor (TFRC) and subsequently alleviates brain damage after strokes by inhibiting neuronal damage and ferroptosis [89]. DMT1 primarily mediates the transport of ferrous iron and heavy metals in the context of systemic iron balance. Nedd4–2 is an E3 ligase that recruits Nedd4 family-interacting protein 1 (Ndfip1) and can effectively mitigate high levels of iron metal toxicity in human neurons via the ubiquitination of DMT1, exerting protective effects [135]. Ferroportin (FPN), recognized as the only iron efflux transporter protein in the human body, is crucial for regulating iron homeostasis in organisms. Exogenous ring finger protein 217 (RNF217) significantly polyubiquitinates FPN and subsequently degrades FPN through the proteasomal and lysosomal pathways, triggering ferroptosis [84]. Iron regulatory proteins mainly include iron regulatory protein 1 (IRP1) and IRP2; both proteins participate in maintaining cellular iron balance [135]. Iron accumulation, neuronal loss, and neurodegenerative motor disorders can result from the absence of the gene encoding IRP2. F-box and leucine-rich repeat protein 5 (FBXL5) act as components of the E3 ligase complex and maintain iron homeostasis by degrading IRP2 through targeted ubiquitination, reducing cell death [80].

Recent studies suggest that the transcription factor (NRF2) could serve as a potential target for regulating ferritinophagy and ferroptosis. The modulation of NRF2-associated signaling pathways significantly attenuates ferritinophagy and ferroptosis after cerebral ischemia–reperfusion. Notably, the Keap1-NRF2-HO-1 signaling pathway plays crucial roles in combating oxidative stress and regulating ferroptosis [136,137]. HACE1, a member of the HECT family of E3 ligases, can activate NRF2. However, the precise role of HACE1 in ferroptosis remains unclear [138].

E3s can regulate ferroptosis by directly targeting proteins and signaling pathways involved in lipid metabolism, such as the ACSL4-mediated ferroptosis pathway. Fbxo10, a subunit of the large E3 ligase family, alleviates ferroptosis, maintains mitochondrial function, and protects neurons from damage by degrading ubiquitinated ASCL4 [81].

Glutathione peroxidase 4 (GPX4) and the cystine/glutamate antiporter system xc^-^ are recognized as crucial negative ferroptosis regulators. SLC7A11, a nonsodium-dependent transporter, facilitates the exchange of intracellular cystine/glutamate, thereby promoting the intracellular synthesis of glutathione (GSH). Utilizing GSH, GPX4 converts the peroxygen bond (L-OOH) of lipid peroxidation into hydroxyl (L-OH) groups, and TRIM family proteins are involved in the regulation of these factors [86]. The interaction between TRIM26 and SLC7A11 facilitates the ubiquitination of the latter [139], whereas TRIM37 affects both GSH contents and GPX4 expression. Moreover, TRIM21, TRIM36, and TRIM59 also interact with GPX4 [140,141]. The intricate relationships among these TRIM family proteins form a crucial foundation for the regulatory network governing ferroptosis.

NCOA4 mediates ferritinophagy, and TRIM7 and TRIM44 can interact with NCOA4 [142,143]. Moreover, NRF2 is crucial for regulating ferroptosis, and TRIM25 facilitates this process by targeting the Keap1-NRF2 pathway. Additionally, HIF-1α serves as a key transcription factor linked to ferroptosis, and its expression is regulated by several TRIM proteins, including TRIM7, TRIM21, TRIM28, and TRIM44 [81].

## 4. Clinical Prospects

The UPS plays a significant role in protein homeostasis, cell cycle control, apoptosis, inflammation, etc. Abnormal UPS activity causes proteasomal dysfunction and abnormal protein accumulation, contributing to neuronal injury and cell death. Thus, targeting the inhibition of the UPS appears to be a promising therapeutic approach. Proteasome inhibitors (PIs)—originally designed to be used in hematological malignancies such as multiple myeloma [105]—can inhibit the proteasome to regulate target protein expression. Recently, they have been shown to participate in ischemic stroke. Treatment with PIs can effectively reduce neuronal and astrocytic degeneration, cortical infarct volume, infarct neutrophil infiltration, and NF-κB immunoreactivity [144]. As an example, carfilzomib, a drug for multiple myeloma therapy, can inhibit proteasomes to reverse BNIP3L degradation and subsequently restore mitophagy in the ischemic brain [137]; the proteasome inhibitor MLN519 reduces infarction and associated neurologic deficits in MACO rats via the inhibition of NF-κB activation, gliosis, and leukocyte infiltration [145]. Unfortunately, few PIs have been reported in the clinical context of ischemic stroke; however, it is worth emphasizing that PIs hold promise as potential therapeutic agents.

E3 ubiquitin ligases can regulate cell cycle progression via ubiquitination, modifying cell cycle proteins and related kinases. During CIRI, cell cycle dysfunction may result in neuronal death. The medications lenalidomide, pomalidomide, and thalidomide—which target specific E3 ligases—may open up new therapeutic prospects [146]. Additionally, targeted protein degradation (TPD)—including the use of proteolysis-targeting chimeras (PROTACs) and molecular glue degraders (MGDs) to degrade proteins—may be another emerging strategy for developing novel therapies [147]. PROTACs or MGDs function by inducing proximity between an E3 ligase and a protein of interest (POI), resulting in the ubiquitination and consequent proteasomal degradation of the POI. TPDs generally exhibit inflated physicochemical properties compared to traditional small molecules, whereas the application of nanoparticles enables the targeted delivery of TPDs. Given the fact that E3 ligases are widely expressed in a wide range of cells, the applications for this technology are promising.

Currently, there is insufficient evidence that E3 ligases exhibit expression preferences at specific sites of cerebral ischemic injury or within specific glial cells. Therefore, the development of cell-specific targeted medications remains challenging. Additionally, although E3 ligases are associated with the degree of pathological damage in ischemic stroke, their use as markers for disease stratification and prognostic assessment remains in the exploratory phase. Nevertheless, targeting E3 ligases in combination with the reperfusion of blood flow in ischemic brain regions remains a promising therapeutic strategy for the future.

## 5. Conclusions and Perspective

The roles of members of the RING, HECT, and RBR E3 ligase families in neuroinflammation, mitophagy, and cell death during CIRI were discussed. Among them, the APC/C complex, a regulator of cell cycle progression, cullins, substrates of Neddylation, FBXO3, the substrate recognition subunit of SKP1-cullin 1-F-box protein (SCF) E3 ligase complexes, NEDD4, a developmental regulatory gene in the CNS, and Parkin are the most promising molecular targets of E3 ligases in CIRI. However, most studies remain limited to basic experimental research. Clinical translation still has its hurdles; further validation of their clinical therapeutic value may need human tissue validation studies. Additionally, for specific E3 ligases, novel tools such as transcriptomics and proteomics can be used to explore their upstream/downstream regulatory factors and reveal new targets. Notably, differences in the expression patterns of E3 ubiquitin ligases between the acute ischemic phase and reperfusion phase are unclear. Further investigation is needed to explore whether E3 ligase activity is altered in different stroke stages.

Currently, despite the promising potential of E3 ligases for treating IS, numerous challenges remain. For example, the E3 ligase family is extensive and continually evolving, with new members likely to emerge that influence associated signaling pathways. Evaluating the regulatory roles of different E3 ligases and precisely modulating a specific E3 will undoubtedly present a formidable challenge.

## Figures and Tables

**Table 1:** Key E3s in CIRI and their molecular mechanisms.

Family	CIRI	E3 Ligase	Molecular Mechanisms	References
RING family	Neuroinflammation	RNF41/RNF56/RNF114	Negatively regulating the NF-κB pathway	[73,74,75]
		RNF121	Positively regulating the NF-κB pathway	[76]
		RNF152	Positively regulating the TLR/IL-1R signaling pathway	[77]
	Necroptosis	RNF216	Suppressing necroptosis through interaction with MLKL	[78]
	Ferroptosis	RNF217	Ubiquitinating FBN to trigger ferroptosis	[79]
		FBOX/FBXL5	Ubiquitinating IRP2 to maintain iron homeostasis	[80]
		FBOX10	Maintaining mitochondrial function and protecting neurons from damage by degrading ubiquitinated ASCL4	[81]
TRIM family	Neuroinflammation	TRIM45/TRIM47	Positively regulating the NF-κB pathway	[32,82,83]
		TRIM 8	In the cytoplasm, positively regulating the NF-κB pathway. In the nucleus, negatively regulating the NF-κB pathway	[84]
		TRIM9	Negatively regulating the NF-κB pathway	[85]
		TRIM62 knockout	Repressing the NLRP3 inflammasome to restrain neuroinflammation	[55]
		TRIM32 knockout	Activating the NRF2 signaling pathway to inhibit oxidative damage	[86]
		TRIM22 knockout	Repressing the NF-κB/NLRP3 axis to restrain neuroinflammation	[87]
		TRIM27 overexpression	Activating the Akt/NRF2/HO-1 pathway to restrain neuroinflammation	[88]
		TRIM16 overexpression	Downregulating Keap1 to enhance NRF2/ARE antioxidant signaling	[89]
		TRIM29	Repressing the NLRP3 inflammasome to restrain neuroinflammation	[90]
	Apoptosis	TRIM21	Inducing apoptosis by targeting the antiapoptotic protein BCL2 through p53	[91]
		TRIM69	Inducing the expression of Bax to promote apoptosis	[56]
		TRIM16	Inducing apoptosis by directly binding to caspase-2 and modulating its activity	[92]
		TRIM27	Positively regulating TNF-induced apoptosis	[93]
		TRIM45 knockout	Inhibiting apoptosis via reversing the overexpression of cleaved caspase-3, caspase-9, and PARP	[33]
HECT family	Ferroptosis	NEDD4L	Ubiquitinating TFRC to inhibit neuronal damage and ferroptosis	[61,94]
		NEDD4-2	Ubiquitinating DMT1 to effectively mitigate high levels of iron metal toxicity	[62]
RBR family	Mitophagy	Parkin	AK4/Parkin/PKM axis prevents cerebral ischemia damage by regulating mitophagy	[95]
Other E3 ligases	Neuroinflammation	TRAF6	Activating RAC1 to promote neuroinflammatory and neuro-oxidative signaling, exacerbating neuronal death	[96,97]
	Necroptosis	CHIP	Negatively regulating necroptosis by increasing the degradation of RIPK1 and RIPK3	[57]
		Pellino3	Preventing the formation of death-induced signaling complexes by targeting RIPK1	[98]
